# Phenotypic Variability Associated with Jagunal Homolog 1 (JAGN1) Deficiency Caused by the c.63G>T Variant

**DOI:** 10.3390/ijms27041735

**Published:** 2026-02-11

**Authors:** Cristina-Loredana Pantea, Mihaela Bataneant, Cristian G. Zimbru, Margit Serban, Maria Puiu, Adela Chirita-Emandi

**Affiliations:** 1 Regional Center of Medical Genetics Timis, Clinical Emergency Hospital for Children “Louis Turcanu”, Part of ERN-ITHACA, 300011 Timisoara, Romania; cristina.pantea@umft.ro (C.-L.P.); adela.chirita@umft.ro (A.C.-E.); 2 Doctoral School, “Victor Babes” University of Medicine and Pharmacy, 300041 Timisoara, Romania; maria_puiu@umft.ro; 3 Second Pediatric Clinic, Clinical Emergency Hospital for Children “Louis Turcanu”, 300011 Timisoara, Romania; 4 Third Pediatrics Clinic, “Victor Babes” University of Medicine and Pharmacy, 300041 Timisoara, Romania; 5 Department of Automation and Applied Informatics, Politehnica University of Timisoara, 300006 Timisoara, Romania; cristian.zimbru@upt.ro; 6 Hematology and Oncology Department, Clinical Emergency Hospital for Children “Louis Turcanu”, 300011 Timisoara, Romania; mserban@spitalcopiitm.ro; 7 Institute for Research and Development in Genomics (ICDG), 020021 Bucuresti, Romania; 8 Department of Microscopic Morphology, Genetics Discipline, Center of Genomic Medicine, “Victor Babeș” University of Medicine and Pharmacy, 300041 Timisoara, Romania

**Keywords:** congenital neutropenia, JAGN1 deficiency, next-generation sequencing

## Abstract

More than 30 distinct genetic entities associated with severe congenital neutropenia (SCN) have been described. SCN has a risk of clonal expansion of mutated hematopoietic cells. Jagunal homolog 1 (JAGN1) deficiency has been described as a genetic cause of SCN and is now estimated to account for approximately 10% of all SCN cases. One prevalent variant in patients with JAGN1 deficiency is NM_032492.4:c.63G>T (p.Glu21Asp). The clinical description and disease evolution study of Romanian patients with JAGN1 deficiency caused by the *JAGN1* c.63G>T variant were performed together with a literature review of similar cases. The clinical characterization of six Romanian patients and nine additional patients reported in the literature with JAGN1 deficiency caused by the c.63G>T variant (40% female) revealed a wide phenotypic spectrum, including: neutropenia (all), severe infections (80%), developmental delay (13%), dental problems such as stomatitis/periodontitis (66%), and short stature (7%). No patient developed malignancy/leukemia during the follow-up period (15 ± 8.1 years). Most patients (93%) had a homozygous variant and consanguineous background, while one had compound heterozygous *JAGN1* variants. The five Romanian patients carrying this homozygous variant, possibly due to a founder effect, had a relatively favorable clinical outcome, with good overall prognosis.

## 1. Introduction

Congenital neutropenia encompasses a group of rare, inborn errors of immunity (IEI) defined by a peripheral absolute neutrophil count of less than 1000 cells/μL blood in the first year of life and less than 1500 cells/μL blood after this age [1,2,3,4,5]. In addition to secondary causes of neutropenia (such as autoimmune neutropenia), there are also multiple IEI associated with congenital forms of neutropenia [3,4,5,6,7]. The International Union of Immunological Societies (IUIS) has categorized congenital neutropenia under the broader category of congenital defects of phagocyte number or function [4,5,8,9,10]. More than 30 distinct genetic entities associated with severe congenital neutropenia (SCN) have been described [3,4,5,6]. Congenital neutropenia is characterized by clinical diversity, ranging from mild or oligosymptomatic presentations to sepsis or fatal infections [3,4,5,11].

Clinical criteria for a probable diagnosis of SCN are: neutropenia below 0.5 × 10^9^/L measured on at least three occasions or neutropenia below 1.0 × 10^9^/L measured on at least three occasions with at least one of the following: deep-seated infection due to bacteria and/or fungi, recurrent pneumonia, buccal and/or genital aphthous lesions or ulcerations, an affected family member, and exclusion of secondary causes of neutropenia [4,5,11].

Hypogammaglobulinemia, lymphopenia, and monocytosis or monocytopenia can be associated with neutropenia [3,4,5,6]. Long-term hematologic complications of congenital neutropenia include an increased risk for myelodysplastic syndrome (MDS) or acute myeloid leukemia (AML) [3,4,5,6,7,12]. Standard care for SCN primarily focuses on infection prevention [4,5,7,13], as well as administration of recombinant granulocyte colony-stimulating factor (G-CSF), and reducing the risk of leukemic transformation; however, certain subtypes manifest additional organ dysfunctions that require specialized management [4,14,15].

According to the 2025 updated release from the International Union of Immunological Societies (IUIS) Expert Committee, variants in more than 67 different genes can cause chronic neutropenia [3,6,8,9,10,15,16,17]. The most common genetic variants associated with SCN include heterozygous germline variants in *ELANE* (typically missense, frameshift, or nonsense variants) and biallelic loss-of-function variants in *HAX1*, such as homozygous nonsense or frameshift changes affecting mitochondrial stability and neutrophil survival (autosomal recessive SCN). Other rare genetic subtypes include X-linked neutropenia due to gain-of-function missense variants in *WAS*, heterozygous dominant-negative missense variants in *GFI1*, heterozygous germline loss-of-function variants (nonsense, frameshift, missense) in *GATA2*, or biallelic missense or frameshift variants in *JAGN1* [8,9,18].

Several smaller studies and case reports have suggested that autosomal recessive JAGN1 deficiency accounts for up to 10% of all patients with SCN worldwide, with varying degrees of disease severity [18,19,20,21,22,23,24,25,26]. Even in cohorts with high consanguinity, the reported prevalence of *JAGN1* variants remains very low—for example, only about 1% (2 out of 216) of people with SCN in the Turkish SCN registry [27]. Certain variants of *JAGN1* are reported in the scientific literature to occur more frequently within specific populations [18,19,20,21,22,24,25,26]. Due to historical isolation, endogamy/consanguinity and genetic drift, some variants, possibly including *JAGN1* NM_032492.4:c.63G>T, have become more common in certain Roma subgroups compared to the general population [28,29,30].

Within the Romanian cohort consisting of 92 children with IEI, variants in the *JAGN1* gene emerged as the most frequent genetic etiology, appearing in approximately 13.5% of molecularly confirmed diagnoses [31]. This high frequency suggests a possible founder effect within these communities. Other genetic causes associated with SCN in the Romanian pediatric cohort with IEI were: *WAS* NM_000377.2:c.172C>A, p.(Pro58Thr), and *GATA2* NM_032638.4:c.1081C>T, p.(Arg361Cys) [31]. Although Romanian-specific nationwide population-based prevalence data for IEI remains undefined, enrollment in the Severe Congenital Neutropenia International Registry (SCNIR) is a clinical priority, ensuring patients benefit from international diagnostic standards and for contributing to the global understanding of SCN.

We aimed to evaluate the *JAGN1* c.63G>T variant in patients from Romania and from the literature to better characterize the variant’s associated phenotype.

## 2. Results

We report four related affected patients (comprising two girls and two boys) and one unrelated patient (male), all of whom are of Roma ethnicity and originate from Romania. From the literature, ten additional patients with the same pathogenic variant were identified, of which one originated from Romania. Thus, a total of 15 patients with a molecularly confirmed *JAGN1* NM_032492.4:c.63G>T variant, present either in the homozygous state or in the compound heterozygous state with another variant, were included in this ana-lysis.

### 2.1. Demographic and Clinical Characteristics

The main clinical and demographic characteristics of the entire cohort (*n* = 15) are summarized in Table 1 and Table 2. Males accounted for 60% of the cohort, while the mean age (±standard deviation) at the time of initial clinical diagnosis was 2.6 ± 2.8 years. The mean age at the last follow-up evaluation was 15 ± 8.1 years, and all patients were alive at the time of data collection, with the oldest individual being 34 years old, some having their own children. The follow-up information was missing for one patient from the Romanian cohort: the unrelated patient has not returned for follow-up, as he is being monitored in another country. Regarding ethnicity and family background, 40% of the patients were identified as Roma, while 86% originated from a consanguineous background. Notably, information on country of origin, ethnicity and consanguinity was not available for seven individuals from the published reports.

### 2.2. Genetic Testing and Molecular Findings

NGS was performed using WES in 60% of patients, WGS in 33%, and a targeted gene panel in 6% (one patient). In 93% of patients, molecular testing identified a homozygous, missense, *JAGN1* NM_032492.4:c.63G>T (p.Glu21Asp) variant, located on chromosome 3p25.3 and classified as pathogenic. In one patient, compound heterozygous *JAGN1* NM_032492.4:c.63G>T (p.Glu21Asp), paternal/c.36del (p.Asp13Thrfs*17), maternal, variants were identified. Thus, the genetic diagnosis of autosomal recessive severe congenital neutropenia type 6 (OMIM 616022) was confirmed in all patients included in this study.

For the five Romanian patients there was a substantial delay in achieving a definitive genetic diagnosis, with a mean delay of 10.5 ± 0.8 years, while for patients in the literature this information was not consistently available.

Genomic analysis of the two related female patients from the Romanian cohort (patients P1 and P2, in Table 1) revealed a secondary heterozygous variant of uncertain significance in *HAX1* (NM_006118.3:c.117_122dup). Despite the theoretical possibility of an additive or modifying phenotypic effect, the clinical course and disease severity for these two patients did not differ significantly in severity from the other patients from this cohort.

### 2.3. Clinical Phenotype and Hematologic Features of the Entire Cohort

The majority of patients had a documented history of severe infections (80%). Recurrent infections were highly prevalent and included: severe deep subcutaneous abscesses, adenophlegmon, tuberculosis, hepatitis, and stomatitis, gingivitis, and periodontal disease, the latter causing premature tooth loss in 20%. Additional syndromic features were present in a fifth of patients, including: short stature (one patient), mild facial dysmorphic features (one patient), strabismus and Stilling–Turk–Duane type 1 syndrome (one patient), global developmental delay (two patients). Other features reported were: meningocele (one patient), recurrent epistaxis (four patients), amelogenesis imperfecta (one patient), obesity (one patient), nephrolithiasis (one patient). Notably, none of the patients in this cohort had skeletal malformations or seizures.

The *JAGN1* c.63G>T variant was associated with phenotypic variability. Neutropenia was identified in all patients, while severe neutropenia (based on the lowest reported absolute neutrophil count) was present in eight out of nine patients (88.8%) for whom complete data were available.

The lowest recorded absolute neutrophil count across the cohort was 223 ± 180.5 cells/μL (with individual values ranging from 43 to 630 neutrophils/μL). The most severely affected patient (P4) presented with 800/μL total leukocytes and only 43/μL neutrophils. Monocytosis was present in all patients. Serum immunoglobulin abnormalities were present in the majority of patients, with 75% showing polyclonal hypergammaglobulinemia (predominantly elevated IgG). All of the patients showed maturation arrest of granulopoiesis on bone marrow smear examination, with hypocellular bone marrow, and a marked deficiency or absence of mature neutrophil granulocytes. Importantly, no patient developed MDS or acute leukemia during the follow-up period (mean 15 ± 8.1 years).

### 2.4. Romanian Cohort: Detailed Hematologic Response and Treatment

**Figure 1:**
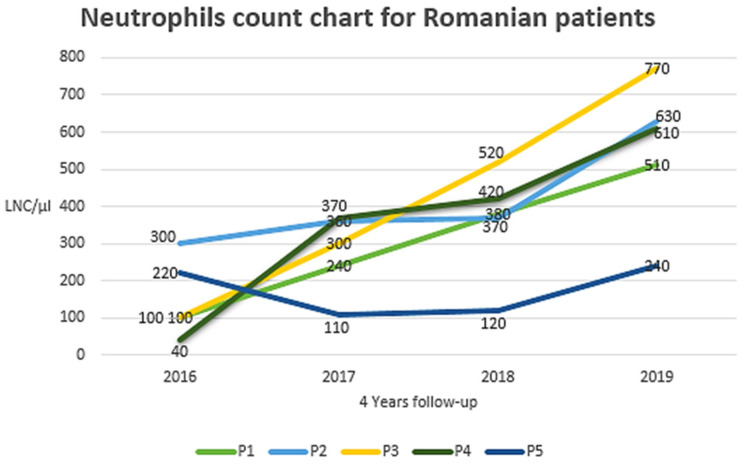
Graphical description of neutrophil counts during G-CSF therapy over 4 years for the 5 Romanian patients with the homozygous c.63G>T p.Glu21Asp *JAGN1* variant. G-CSF—Granulocyte Colony-Stimulating Factor, LNC—Lowest neutrophil count (/µL), Romanian cohort (P1–P5) are also presented in detail in Table 2.

In the Romanian cohort, the maximum monocyte count recorded during G-CSF therapy was 5260 cells/µL, with a normal reference range between 90 and 860 cells/µL. Figure 1 illustrates the lowest neutrophil count (LNC/µL) for the five Romanian patients (P1–P5) over a four-year follow-up period. Recombinant human granulocyte colony-stimulating factor (GM-CSF) was administered subcutaneously once daily, starting at 6–13 µg/kg/day, with the dose escalated in stepwise increments ultimately up to 18–20 µg/kg/day, based on clinical response and absolute neutrophil count monitoring. Most patients (P1, P2, P3, P4) showed a general upward trend in their neutrophil counts over the four-year observation period during the treatment. Patient P5, however, experienced the poorest response, with absolute neutrophil counts not exceeding 240/µL.

### 2.5. Combined Cohort: G-CSF Treatment and HSCT

All patients received G-CSF treatment, with a median daily dose of 13 µg/kg body weight, although most patients were non-responsive or demonstrated poor clinical response. The mean age at G-CSF treatment initiation was 8.2 ± 4.93 years. Human leukocyte antigen (HLA) typing was performed for transplant evaluation, and 44.4% of patients from the entire cohort ultimately underwent hematopoietic stem cell transplantation (HSCT) as definitive curative therapy.

### 2.6. Variant Frequency Analysis

No carriers of the *JAGN1* variant were identified in our in-house database of Romanian patients (2000 people tested for various genetic disorders with WES, WGS or gene panels that include *JAGN1* gene). The variant SNV:3-9890785-G-T (GRCh38) has a frequency in Gnomad v4.1.0. of f = 0.000001864 (three European individuals).

## 3. Discussion

SCN caused by the *JAGN1* c.63G>T variant represents a multisystem, clinically heterogeneous disorder characterized by early-onset severe neutropenia, recurrent and severe infections, and extra-hematopoietic manifestations.

In this report we describe the largest group to date of patients carrying the homozygous *JAGN1* NM_032492.4:c.63G>T (p.Glu21Asp) variant, comprising five newly characterized individuals of Roma ancestry together with ten previously published reports [18,19,21,22,23,24,25,26,27].

The genetic diagnosis of patients P1–P5 with *JAGN1* homozygous variant NM_032492.4 c.63G>T, p.(Glu21Asp) was reported previously in our nationwide genetic study [31]. The present report expands on these findings by providing additional phenotypic data and longitudinal follow-up not included in the original publication.

The *JAGN1* variants are inherited in an autosomal recessive pattern. Ninety-three percent of patients from our cohort inherited the same alleles from both parents. This specific missense variant is located on chromosome 3p25.3 and is classified as pathogenic. A distinct inheritance pattern was observed in one individual (P8) who presented with compound heterozygous variants. This patient inherited the c.63G>T (p.Glu21Asp) missense variant from their father and the c.36del (p.Asp13Thrfs*17) frameshift variant from the mother. The presence of these *JAGN1* variants on both alleles underlies the genetic basis of the condition.

Affected individuals showed early symptom onset. A substantial delay in genetic diagnosis was observed for the Romanian patients, due to limited access to NGS testing, especially for those born in previous decades, when such testing was not widely available or affordable. Other countries, particularly those in developing regions, may have similar issues; however, this information was not available in the published literature for this group of patients. Nonetheless, these findings underscore the importance of early molecular testing in children presenting with unexplained congenital neutropenia. WES and WGS have emerged as powerful approaches for identifying novel candidate genes contributing to neutropenia [32].

A particularly notable feature of this cohort is the strong familial pattern, with 40% of patients reporting similarly affected relatives and 86% originating from consanguineous family backgrounds, where this information was available. Together with the over-representation of Roma ethnicity (40% of the cohort where ethnicity data were available), these findings suggest the hypothesis that the c.63G>T variant may represent a founder mutation within this population [18,19,20,21,22,23,24,25,26,28,29]. The mean run of homozygosity length of the Roma groups is significantly higher than the mean of the non-Roma, reflecting the unique demographic history of this ethnic group [29]. Ethnicity is an important factor in genetic analyses, so a population-specific approach in both clinical and scientific settings has important implications for clinical practice. However, the study design does not allow us to demonstrate this hypothesis, as ethnicity and consanguinity data were unavailable for several previously reported individuals. Furthermore, formal haplotype analysis will be needed to conclusively establish whether all or most individuals carrying the c.63G>T variant share a common ancestral haplotype, thereby confirming its status as a founder variant [3,4,5,6,7,12,21,23,27].

The identification of compound heterozygosity in one patient (c.63G>T/c.36del) demonstrates the allelic heterogeneity of JAGN1 deficiency and underscores the importance of comprehensive genetic testing that includes sequencing of all coding exons and splice junctions, as well as copy number variant analysis when appropriate (negative results).

Across all 15 patients included in this analysis, the clinical phenotype was variable, despite the presence of the same underlying variant in the vast majority (93%) of individuals. Neutropenia was universal, and nearly all evaluable patients exhibited severe neutropenia. Bone marrow examination consistently revealed marked maturation arrest of granulopoiesis, accompanied by hypocellular marrow and near-complete absence of mature neutrophils. These findings are in agreement with previous reports and are consistent with the known pathophysiology of JAGN1 deficiency [18,19,20,21,22,23,24,25,26,28]. The erythroid and megakaryocytic lineages were generally preserved; anemia or thrombocytopenia, when present, tended to be mild or secondary to other factors [22,24]. Neutrophil count variations in SCN are primarily determined by the specific genetic variant, its impact on neutrophil differentiation and survival, and the degree of responsiveness to growth factor therapy.

Hypergammaglobulinemia and monocytosis were recurrent laboratory findings in JAGN1 deficiency in our cohort and likely reflect chronic immune activation secondary to persistent or recurrent infections. Monocytosis may also appear or become more pronounced following initiation of G-CSF treatment in some patients [33]. The universal presence of monocytosis is a characteristic feature that may help distinguish JAGN1 deficiency from other genetic causes of SCN, such as GATA2 deficiency, which typically presents with monocytopenia in addition to neutropenia [6].

In the context of autosomal recessive neutropenia, the presence of multiple gene variants related to myeloid development or function suggests a potential shift toward an oligogenic inheritance model, wherein secondary variants may modify the primary phenotype or contribute to phenotypic variability. While autosomal recessive neutropenia typically results from biallelic (loss-of-function) defects in a single gene—such as *HAX1* or *G6PC3*—additional heterozygous variants can exert a “double hit” effect on the myeloid maturation pathway or cellular stress response. For instance, the HCLS1-associated protein X-1 (HAX1 protein) is essential for maintaining mitochondrial membrane potential and preventing premature apoptosis in neutrophil precursors [33]. A secondary variant in this gene, even if heterozygous, could theoretically exacerbate the unfolded protein response, further sensitize cells to apoptotic triggers, or reduce the threshold for activation of cell death pathways. However, the clinical manifestation of these additive effects might be negligible or undetectable in practice, considering the primary recessive variant causes complete loss of function or reaches a critical biological threshold, leading to no measurable increase in clinical severity. Despite this, documenting these modifiers might be important for understanding phenotypic variability, as they may influence non-hematological symptoms or the long-term risk of malignant transformation to leukemia [17,34,35].

Severe, recurrent, and atypical infections were highly prevalent in this cohort. These encompassed deep soft-tissue abscesses, adenophlegmon, tuberculosis, hepatitis, and extensive oral infections. Periodontal disease, characteristic of severe congenital neutropenia, was observed in 20% of patients carrying the p.Glu21Asp variant. Extra-hematopoietic manifestations were similarly heterogeneous and included short stature, mild facial dysmorphism, neurodevelopmental delay, and various organ-specific abnormalities (meningocele, recurrent epistaxis, obesity, nephrolithiasis). This syndromic spectrum suggests that *JAGN1* may play broader biological roles beyond granulopoiesis, potentially impacting bone, dental tissues, metabolism, and platelet or endothelial cell functionality. The described cases confirm that oral mucosal lesions in patients with severe congenital neutropenia (SCN) are frequently infectious and the most characteristic symptoms are gingivitis and rapid periodontal destruction that cannot be controlled by local treatments. In addition to its well-known role in neutrophil maturation, the *JAGN1* gene may also impact platelet function or clotting factors, potentially leading to bleeding disorders in affected individuals. Bleeding manifestations, which we observed in several people in the Romanian cohort, have also been described in the literature [18,19,20,21,22,23,24,25,26,28].

A case report and literature review published by Thomas S et al. in 2023 focuses on a patient with autosomal recessive JAGN1 deficiency who experienced bleeding manifestations [18]. Notably, the presence of extra-hematopoietic manifestations did not correlate with the severity of neutropenia or infectious burden [23,32].

Despite the disease’s severity, neither MDS nor AML was identified in this cohort, during a mean follow-up period of 15 years, contrasting with other forms of SCN in which leukemic transformation is more frequent [3,4,5,6,7,12,17,21,22,27]. Larger cohorts and longer follow-up will be required to clarify the true long-term hematologic malignancy risk in JAGN1 deficiency. Exploring the mechanisms of somatic genetic rescue presents a promising avenue for potentially preventing or delaying leukemia transformation in susceptible individuals.

It is noteworthy that the another *JAGN1* variant, c.35_43delCCGACGGCA (p.Thr12_Gly14), has been reported in association with a syndromic spectrum and progression to malignancy (AML) at 18 years of age [21].

To provide broader context, a prospective study of 374 individuals with SCN from the Severe Chronic Neutropenia International Registry reported a 15-year cumulative incidence of 22% for MDS/AML among patients receiving G-CSF therapy [35]. Similarly, an analysis of the French Neutropenia Registry among patients reported a cumulative incidence of MDS/AML of 8.1% at 20 years, including both patients who received and did not receive G-CSF [17]. An elevated risk of developing hematological abnormalities and malignant transformation has been reported mainly for patients with *ELANE*, *HAX1*, and *SBDS* pathogenic variants, however, it has also been observed in individuals with *GATA2*, *G6PC3*, and *CLPB* variants [4]. To our knowledge, disease progression to MDS and/or AML has only rarely been reported in patients with JAGN1 deficiency [4,22]. In a cohort with SCN caused by *JAGN1* variants, only one patient developed MDS/AML at age 18 years, yet none with the c.63G>T variant [21].

In a recent retrospective multicenter study conducted by the European Society for Blood and Marrow Transplantation (EBMT) and Inborn Errors Working Party (IEWP) in close collaboration with the European Society for Immunodeficiencies (ESID) and the French Neutropenia Registry, eight patients with the p.Glu21Asp variant were identified and characterized [21,36]. Among these eight individuals, four (50%) exhibited dental anomalies (hypodontia, enamel hypoplasia, and delayed tooth eruption). Concerning ethnic and geographic demographics, the study categorizes all eight p.Glu21Asp lineages as of “Middle Eastern” origin, so this variant may represent a regional founder variant within the Middle Eastern population.

In a cohort of 74 patients with SCN, Boztug et al. in 2014 identified 9 additional individuals from 8 distinct families carrying homozygous *JAGN1* variants [22]. The clinical presentation in these patients was largely similar to those with pathogenic variants in *ELANE* or *HAX1* associated with non-syndromic SCN. Notably, many patients with JAGN1 deficiency in this cohort exhibited suboptimal response to G-CSF therapy, and some experienced severe osteoarticular pain that limited its use [22].

Consistent with prior reports, response to G-CSF was limited, with most patients showing partial or absent improvement. The reasons remain unclear. This poor response may be attributed to the role of *JAGN1* in the early secretory pathway and its necessity for proper G-CSF receptor-mediated signaling. Prior studies have shown that 5–10% of SCN patients overall do not respond to G-CSF, although predictive biomarkers are lacking [4,5,13]. In the Romanian patients, GM-CSF was given in all children, however, it did not provide substantial clinical benefit.

According to Castagnoli et al. (2019), SCN represents one of the established indications for hematopoietic stem cell transplantation (HSCT) in patients with IEI [14]. Given the severity and frequency of infections, the persistence of neutropenia and non-responsiveness to G-CSF treatment, HSCT was performed in 44% of the evaluable patients, a proportion similar to that reported in the multicenter European Society for Blood and Marrow Transplant (EBMT)/European Society for Immunodeficiencies (ESID) retrospective study [21,36]. HSCT remains the only curative treatment option for SCN [15]. Current IEI/SCN guidelines recognize JAGN1 deficiency as an indication for transplantation in G-CSF non-responders, in patients who are partial responders but require prohibitively high doses of G-CSF or those with significant organ involvement. HSCT has the potential to remedy SCN; however, its efficacy is constrained by the scarcity of compatible donors and is accompanied by the potential for graft-versus-host disease and infectious mortality [14,15,37]. Such limitations serve as compelling incentives to explore and develop alternative therapeutic strategies for SCN, and genetic investigations have paved the way for the identification of prospective novel therapeutic agents [6].

Several published reports—including our current findings—show that dental anomalies, syndromic features, and neurodevelopmental findings may occur even among individuals sharing identical *JAGN1* pathogenic variants [11,18,19,20,21,22,23,24,25,26]. These observations highlight the need for genotype–phenotype studies as more patients with JAGN1 deficiency and other rare genetic disorders are identified through newborn screening programs, targeted carrier screening in high-risk populations, and exome/genome sequencing. Additional molecular variants (either rare variants in *JAGN1* or polygenic) could explain a substantial proportion of the observed phenotypic variability, indicating that additional factors modulate disease expression. The phenotypic variability observed even among patients with the identical homozygous c.63G>T variant suggests the influence of additional genetic modifiers or environmental factors.

Analyzing the single nucleotide polymorphism (SNP) haplotype background represents a powerful approach to support the hypothesis that *JAGN1* p.Glu21Asp is a founder mutation. However, further studies and further accumulation of additional patients will be needed.

An article from 2025 [37] describes a new autosomal recessive form of *COPZ1*-associated SCN, where some patients exhibit monocytosis and hypergammaglobulinemia, resembling findings in JAGN1 deficiency (e.g., c.63G>T variant). Both *COPZ1* and *JAGN1* mediate ER–Golgi trafficking, and their dysfunction triggers monocyte activation and hypergammaglobulinemia [37].

Another study from 2025 [38] presents the results of an extensive nationwide genetic screening that identified multiple genetic variants associated with SCN, including a new homozygous variant in *JAGN1* [38]. It highlighted the genetic and phenotypic diversity of patients with SCN, underscoring the importance of genetic testing for identifying cases with atypical or syndromic clinical manifestations [38].

Standard clinical management for *JAGN1*-related SCN prioritizes G-CSF; however, for patients failing this regimen or presenting with severe infection pathology, HSCT remains the only definitive curative pathway. To address these limitations, research is currently targeting the mechanisms of *JAGN1*-mediated G-CSF resistance, with a focus on ER stress modulators as a novel therapeutic strategy. Additionally, gene therapy is being explored as a permanent intervention. Mesenchymal stem cell (MSC)-based therapies could could help manage neutropenia-related inflammation through enhanced immune regulation [39].

Mesenchymal stem cells are bone marrow stromal cells that help control myeloid differentiation and the maturation of neutrophils. MSCs produce essential cytokines and growth factors, including G-CSF, which promotes neutrophil proliferation and maturation [39]. While the 2024 article on MSCs [39] highlights the ability of MSCs to modulate immune responses during active infections, this potential is particularly relevant for patients with JAGN1 deficiency caused by the c.63G>T variant. Given the significant phenotypic variability and susceptibility to severe infections seen in these patients, MSC-based therapies could theoretically offer a secondary layer of immune regulation to mitigate the inflammatory complications associated with their underlying neutropenia [39].

Proper neutrophil development relies on MAPK signaling triggered by the G-CSF receptor [40]. However, the *JAGN1* c.63G>T variant interferes with this process by causing G-CSFR glycosylation defects and reducing its presence on the cell membrane. Given that MAPK signaling is essential for orchestrating immunity and sepsis responses [40], the diminished signaling capacity inherent in JAGN1 deficiency provides a molecular basis for the increased infection susceptibility and phenotypic diversity observed in affected individuals.

## 4. Materials and Methods

### 4.1. The Romanian Cohort

In a preceding retrospective analysis of 92 children with suspected IEI at a tertiary center in Romania, the *JAGN1* c.63G>T variant emerged as a notable cause of neutropenia [31]. This homozygous variant was identified in five patients (P18–P22), initially described in brief by Pantea et al. [31]. Here, we expand upon those findings by defining the detailed clinical phenotype associated with this variant [31].

Patient evaluation included assessment of gender, ethnicity, parental consanguinity, symptoms, age at symptom onset, age at last reported follow-up, infection history, non-infectious manifestations, genetic testing performed, genotype, laboratory findings (lymphocyte subsets, immunoglobulin levels and specific antibodies), and treatment outcomes. Severe infections included abscesses, septicemia, pneumonia, and otomastoiditis. All Romanian patients underwent genetic testing. NGS was performed in a research setting using WES or WGS according to methodology published elsewhere [31].

Classification followed the American College of Medical Genetics and Genomics and the Association for Molecular Pathology (ACMG/AMP) guidelines [41], categorizing variants as pathogenic (P), likely pathogenic (LP), variant of uncertain significance (VUS), likely benign (LB), or benign (B). Findings were ultimately reported as disease-causing, negative, or uncertain based on the variant classification and the associated inheritance pattern [41,42]. For the management of data, clinical and genomic information was consolidated and anonymized within a Microsoft Excel database for subsequent analysis.

### 4.2. Genomic DNA Isolation and Quality Control

In all participating patients, genomic deoxyribonucleic acid (gDNA) was extracted from whole blood samples using the MagCore® Automated Nucleic Acid Extractor in conjunction with the MagCore® Genomic DNA Whole Blood Kit (RBC Bioscience, New Taipei City, Taiwan). All extraction procedures strictly adhered to the manufacturer’s standardized protocols to ensure appropriate-purity yields [31]. The quality and concentration of the extracted gDNA were verified through a dual-quantification approach. Purity was assessed via UV–Vis absorbance using the BioTek Epoch Spectrophotometer (Ag-ilent Technologies Inc., Santa Clara, CA, USA), while concentration measurements were obtained using the Qubit® double-stranded DNA (dsDNA) High Sensitivity (HS) Assay Kit (Invitrogen, Carlsbad, CA, USA), as previously presented [31]. 

### 4.3. Sequencing Platforms and Library Preparation

Genetic investigations were performed at Novogene (Cambridge, United Kingdom) using Illumina short-read NGS technologies [31]. The selection between WES and WGS was determined by the clinical suspicion and the availability of resources (research project available). These investigations were provided to the patients at no cost. For all samples, DNA fragmentation was achieved through sonication. WES libraries were prepared using the SureSelect Human All Exon V6 kit (Agilent Technologies Inc., Santa Clara, CA, USA), targeting all protein-coding exons and flanking exon–intron boundaries (±20 base pairs), and were sequenced to an average depth of 100X. For WGS, libraries were constructed using the NEB Next® Ultra™ DNA Library Prep Kit (New England Biolabs, Hitchin, United Kingdom) and sequenced to an average coverage of 30X. Both methods were executed on the Illumina Novaseq 6000 PE150 high-throughput platform (Illumina, San Diego, CA, USA), as previously shown [31].

### 4.4. Bioinformatics Pipeline and Variant Interpretation

Bioinformatics processing and data interpretation were performed at the Center of Genomic Medicine, a research laboratory within the “Victor Babeș” University of Medicine and Pharmacy, Timisoara, Romania. End-to-end bioinformatics algorithms were implemented, beginning with base alignment to the GRCh38 reference genome and followed by the primary filtering of low-quality reads and potential sequencing artifacts [31]. The DRAGEN v4.0 Illumina software (Illumina, San Diego, CA, USA) was utilized for the alignment and identification of variants. Variant interpretation was conducted through a comprehensive synthesis of population, segregation, computational, functional, and phenotypic data, as previously reported [31].

### 4.5. Clinical Classification and Statistical Analysis 

Human genetic variation was analyzed using several global databases, including the Databases of human genetic variation used, included the Genome Aggregation Database (gnomAD v4; https://gnomad.broadinstitute.org/, accessed on 28 December 2025) and ClinVar (https://www.ncbi.nlm.nih.gov/clinvar, accessed on 28 December 2025), and the Clinical Genome Resource (ClinGen; https://www.clinicalgenome.org/, accessed on 28 December 2025) for expertly curated reports genes-disease associations [41]. Potential impacts on alternative splicing were investigated using SpliceAI and Human Splicing Finder, while WGS-specific data were analyzed using MOON software (www.diploid.com/moon, accessed on 28 December 2025). Variants were prioritized if they demonstrated a minor allele frequency (MAF) of less than 1% in gnomAD or were previously documented as disease-causing in the Human Gene Mutation Database (HGMD®) [41].

### 4.6. Literature Search

A targeted literature review was conducted in order to enable a better understanding of the clinical findings in patients with JAGN1 deficiency caused by the *JAGN1* c.63G>T (p.Glu21Asp) variant. A systematic search was performed in PubMed, Scopus and Google Scholar using the combinations of the following keywords: “*JAGN1*”, “c.63G>T”, “p.Glu21Asp”, “severe congenital neutropenia”. Boolean operators (“AND”, “OR”) helped refine the search. Both homozygous and compound heterozygous statuses of genetic variants were included.

Information on patients’ gender, ethnicity, parental consanguinity, phenotypic presentation, age at clinical diagnosis, age at last reported follow-up, country of origin, genetic testing performed, genotype, laboratory investigation and treatment outcomes was systematically collected and summarized. Individuals with missing data were omitted from the denominator when calculating the prevalence of clinical signs and symptoms in the descriptive analysis (Table 1 and Table 2).

## 5. Conclusions

The SCN caused by the *JAGN1* c.63G>T (p.Glu21Asp) variant represents a multisystemic, clinically heterogeneous disorder characterized by early-onset, severe neutropenia, recurrent or severe infections, extra-hematopoietic manifestations, and suboptimal or absent responsiveness to G-CSF therapy. The long-term risk associated with malignant transformation appears to be lower than in other forms of SCN, though continued surveillance remains essential. The five Romanian patients carrying this homozygous variant had a relatively favorable clinical outcome, with good overall prognosis. The case series contributes to understanding the diverse clinical outcomes associated with *JAGN1* variants and emphasizes the importance of advanced genetic sequencing techniques in diagnosing these patients accurately. Evidence derived from our cohort, in conjunction with existing literature, suggests that this variant may be enriched—or potentially act as a founder variant—within the Roma ethnic group, warranting further genetic studies, including haplotype analysis.

Limitations: This study is limited by unequal follow-up. Missing demographic data (ethnicity, consanguinity) in previously published individuals restricts conclusions regarding founder effects. Additionally, detailed functional studies of the c.63G>T variant and its effects on JAGN1 protein function were not performed in this study. Finally, the small number of documented patients globally limits the ability to draw robust genotype–phenotype correlations and may introduce ascertainment bias, as more severe or syndromic presentations are preferentially reported.

## Figures and Tables

**Table 1:** Genetic findings in patients with *JAGN1* c.63G>T (p.Glu21Asp) variants from our cohort and selected cases from the literature.

Patient	Gender	Age at Clinical Diagnosis (Years)	Country/Ethnicity	Consanguineous	Genetic Test Performed	Variant *JAGN1*, NM_032492.4 c.63G>T	PMID
Summary (15 children)	60% M	2.6 ± 2.8 y	40% R	86%	60% WES, 33% WGS	93.3% hom	
1	F	0.3	Romania/Roma	Y	WGS	hom	40442269 (P18)
2	F	0.2	Romania/Roma	Y	WGS	hom	40442269 (P19)
3	M	0.2	Romania/Roma	Y	WGS	hom	40442269 (P20)
4	M	0.4	Romania/Roma	Y	WGS	hom	40442269 (P21)
5	M	7	Romania/Roma	Y	WES	hom	40442269 (P22)
6	M	0.8	Romania/Roma	N	600-gene panel	hom	30044346, 39775668 (P19)
7	F	3	NA	NA	WES	hom	39286252 (P1)
8	F	0.3	NA	N	WES	het/het *	39286252 (P2)
9	F	NA	Albania/Albanian	Y	WGS	hom	25129144 (P12), 39775668 (P37)
10	M	5	NA	NA	WES	hom	33206996, 39775668 (P17)
11	M	5	NA	NA	WES	hom	33206996, 39775668 (P16)
12	M	8	NA	NA	WES	hom	39775668
13	M	NA	NA	NA	WES	hom	39775668
14	M	NA	NA	NA	WES	hom	39775668
15	F	NA	NA	NA	WES	hom	39775668

F—female; het—heterozygous; hom—homozygous; *—*JAGN1* NM_032492.4:c.63G>T (p.Glu21Asp), paternal/c.36del (p.Asp13Thrfs*17), maternal; M—male; N—no; NA—Not available; P—patient; PMID—PubMed Identifier; R—Roma ethnicity; WES—Whole Exome Sequencing; WGS—Whole Genome Sequencing; Y—yes; y—years.

**Table 2:** Clinical details for patients with *JAGN1* c.63G>T (p.Glu21Asp) variants from our cohort and selected cases from the literature.

Patient	Age at Clinical Diagnosis (Years)	Age at Last Follow-Up (Years)	Severe Infections	Infection History	Developmental Delay	Other Clinical Features	LNC/µL	Serum Immunoglobulin Levels	Age (Years) at GM-CSF Treatment	HSCT	PMID
Summary (15 children)	2.6 ± 2.8 y	15 ± 8.1 y	60% Y	66% dental issues, 50% ENT issues	13%	80%	223 ± 180.5	75% hyper-Ig	8.2 ± 4.9	26%	
1	0.3	17	Y	Otomastoiditis, periodontitis	N	Recurrent epistaxis	240	↑IgG, IgA, IgM	10	N	40442269 (P18)
2	0.2	16	N	Periodontitis	N	Recurrent epistaxis	100	↑IgG	8	N	40442269 (P19)
3	0.2	15	N	Periodontitis	N	Recurrent epistaxis	100	↑IgG, IgA	7	N	40442269 (P20)
4	0.4	14	N	No severe infections	N	Recurrent epistaxis	43	↑IgG, IgA	8	N	40442269 (P21)
5	7	23	Y	Adenophlegmon, tuberculosis, hepatitis, stomatitis, abscesses	N	Obesity	50	↑IgG, IgA	7	N	40442269 (P22)
6	0.8	6	Y	Otitis, abscesses, gingivostomatitis, candidiasis, cellulitis, pneumonia	Y	Dysmorphic face, convergent monocular strabismus, cardiac abnormalities, Stilling–Turk–Duane type 1 syndrome	630	↑IgA	0.6	Y (2 y)	30044346, 39775668 (P19)
7	3	22	Y	Gangrene-induced sepsis, otitis, urosepsis, mastoiditis, pneumonia, gingivitis	N	NA	200	↓IgG	17	Y (20 y)	39286252 (P1)
8	0.3	2	Y	Pneumonia, oral thrush	N	Triangular face	237	↓IgG	0.4	Y (0.8 y)	39286252 (P2)
9	NA	16	Y	Upper respiratory tract infections, pneumonia, skin abscess	Y	Amelogenesis imperfecta, short stature	408	NA	NA	NA	25129144 (P12), 39775668 (P37)
10	5	34	Y	Recurrent bacterial infections, otitis media, bronchitis, perianal abscesses, periodontitis, tooth loss	N	Nephrolithiasis	NA	NA	NA	NA	33206996, 39775668 (P17)
11	5	20	Y	Recurrent, severe bacterial infections, otitis, bronchitis, sinusitis, pneumonia, chronic gingivitis, mouth ulcers, periodontitis, tooth loss	N	NA	NA	NA	NA	NA	33206996, 39775668 (P16)
12	8	11	Y	Pneumonia, skin infections, periodontitis, tooth loss	N	NA	NA	NA	NA	Y (1.6 y; 2.5 y)	39775668
13	NA	6	N	No severe infections	N	NA	NA	NA	NA	NA	39775668
14	NA	19	N	Chronic otitis media	N	Meningocele	NA	NA	NA	NA	39775668
15	NA	4	N	No severe infections	N	NA	NA	NA	NA	NA	39775668

ENT—ear, nose, throat (otorhinolaryngology); GM-CSF—Granulocyte–Macrophage Colony-Stimulating Factor; HSCT—hematopoietic stem cell transplantation; Ig—immunoglobulin; LNC—Lowest neutrophil count; µL—microliter; N—no; NA—Not available; P—patient; PMID—PubMed Identifier; Y—yes; y—years; ↑—high level; ↓—low level.

## Data Availability

The data presented in this study are available on request from the corresponding author. The data are not publicly available due to privacy restrictions, as they contain sensitive genetic and clinical information that could compromise patient confidentiality.

## Abbreviations

The following abbreviations are used in this manuscript:

AML	Acute myeloblastic leukemia
ANC	Absolute neutrophil count
EBMT	European Society for Blood and Marrow Transplantation
ESID	European Society for Immunodeficiencies
G-CSF	Granulocyte colony-stimulating factor
HSCT	Hematopoietic stem cell transplantation
IEI	Inborn error of immunity/Inborn errors of immunity
JAGN1	Jagunal homolog 1
MDS	Myelodysplastic syndrome
NGS	Next generation sequencing
SCN	Severe congenital neutropenia
WES	Whole exome sequencing
WGS	Whole genome sequencing
